# The Interplay of Work, Digital Health Usage, and the Perceived Effects of Digitalization on Physicians’ Work: Network Analysis Approach

**DOI:** 10.2196/38714

**Published:** 2022-08-17

**Authors:** Petra Saukkonen, Marko Elovainio, Lotta Virtanen, Anu-Marja Kaihlanen, Janna Nadav, Tinja Lääveri, Jukka Vänskä, Johanna Viitanen, Jarmo Reponen, Tarja Heponiemi

**Affiliations:** 1 Finnish Institute for Health and Welfare Helsinki Finland; 2 Department of Psychology and Logopedics Faculty of Medicine University of Helsinki Helsinki Finland; 3 Infectious Diseases and Meilahti Vaccine Research Center MeVac Inflammation Center University of Helsinki and Helsinki University Hospital Helsinki Finland; 4 Department of Computer Science Aalto University Espoo Finland; 5 Finnish Medical Association Helsinki Finland; 6 Research Unit of Medical Imaging Physics and Technology University of Oulu Oulu Finland; 7 Medical Research Centre Oulu Oulu University Hospital and University of Oulu Oulu Finland

**Keywords:** network analysis, mixed graphical model, physicians, health care digitalization, digitalization of work, work in transformation, digital health

## Abstract

**Background:**

In health care, the benefits of digitalization need to outweigh the risks, but there is limited knowledge about the factors affecting this balance in the work environment of physicians. To achieve the benefits of digitalization, a more comprehensive understanding of this complex phenomenon related to the digitalization of physicians’ work is needed.

**Objective:**

The aim of this study was to examine physicians’ perceptions of the effects of health care digitalization on their work and to analyze how these perceptions are associated with multiple factors related to work and digital health usage.

**Methods:**

A representative sample of 4630 (response rate 24.46%) Finnish physicians (2960/4617, 64.11% women) was used. Statements measuring the perceived effects of digitalization on work included the patients’ active role, preventive work, interprofessional cooperation, decision support, access to patient information, and faster consultations. Network analysis of the perceived effects of digitalization and factors related to work and digital health usage was conducted using mixed graphical modeling. Adjusted and standardized regression coefficients are denoted by b. Centrality statistics were examined to evaluate the relative influence of each variable in terms of node strength.

**Results:**

Nearly half of physicians considered that digitalization has promoted an active role for patients in their own care (2104/4537, 46.37%) and easier access to patient information (1986/4551, 43.64%), but only 1 in 10 (445/4529, 9.82%) felt that the impact has been positive on consultation times with patients. Almost half of the respondents estimated that digitalization has neither increased nor decreased the possibilities for preventive work (2036/4506, 45.18%) and supportiveness of clinical decision support systems (1941/4458, 43.54%). When all variables were integrated into the network, the most influential variables were purpose of using health information systems, employment sector, and specialization status. However, the grade given to the electronic health record (EHR) system that was primarily used had the strongest direct links to faster consultations (b=0.32) and facilitated access to patient information (b=0.28). At least 6 months of use of the main EHR was associated with facilitated access to patient information (b=0.18).

**Conclusions:**

The results highlight the complex interdependence of multiple factors associated with the perceived effects of digitalization on physicians’ work. It seems that a high-quality EHR system is critical for promoting smooth clinical practice. In addition, work-related factors may influence other factors that affect digital health success. These factors should be considered when developing and implementing new digital health technologies or services for physicians’ work. The adoption of digital health is not just a technological project but a project that changes existing work practices.

## Introduction

### Background

Digital transformation is rapidly changing the health care sector, and the COVID-19 pandemic has accelerated this transition to digital solutions [1]. The digital transformation of health care is expected to enhance health outcomes by improving person-centered care and self-management, data-driven treatment decisions, and medical diagnoses as well as creating more evidence-based knowledge, skills, and competencies for professionals to support health care delivery [2]. Physicians are one of the most important stakeholders in health care, and they have the potential to shape this change for the benefit of clinical care [3].

The digital transformation of workplace can be defined as a phenomenon in which new technologies significantly change the way employees perform tasks and processes, their social relationships, and subsequently their overall workplace experience [4]. Indeed, physicians have seen digital health as a dynamic facet of new ways of working [5]. Digital health is the field of knowledge and practice related to the development and use of digital technologies to improve health [2]. The broad scope of digital health includes categories such as health information systems (HISs; including, eg, electronic health record [EHR] systems and clinical decision support systems [CDSSs]) as well as telemedicine, wearable devices, mobile health, and personalized medicine [6].

Digital health can provide additional work processes next to existing ones or completely replace current processes [7]. It also changes the culture toward shared decision-making and the democratization of care [8]. Patients are suggested to no longer be just customers but active participants in their own care processes [5,8]. Digital health can empower patients to advocate for themselves, take control of their care, and make better-informed decisions about their health [9-11]. In a variety of settings, digital interventions can be effective in both preventing and treating disease [12-17]. In addition, digital health appears to impact interprofessional cooperation [18-23]. EHRs influence cooperation by facilitating access to patient information and data sharing between different stakeholders and hospitals [21,24,25]. There is also a need for effective cooperation between information technology professionals and physicians to improve the quality and implementation of HISs [26]. Participation in development may also increase one’s sense of control over work [27]. Technology such as CDSSs can support physicians by minimizing errors [28], improving the accuracy of physician diagnoses [29] and outcomes [28,30,31], and increasing efficiency [29,32]. Physicians have described the greatest benefits of digitalization in terms of care quality, readability, and ease of access to patient data [33,34]. In addition, digitalization has been shown to support collaboration, decision-making, and continuous learning [5], and it has been associated with improved job satisfaction and work-life balance [35].

However, the digital transformation of health care is a highly multifaceted issue [7]. Physicians have expressed concerns about the impact of digitalization on information overload and ambiguity, interaction with patients, privacy issues, disruptions to workflows, and increasing workloads [5,34,36-39]. The digitalization of work has also been found to be associated with the stress levels of physicians [40,41]. Dissatisfaction has been particularly associated with the implementation of new EHRs [42-45] and the subsequent transition period [46].

Reports on the impact of digitalization on work performance have been inconclusive [34]. Digitalization potentially affects and is affected not only by the characteristics of the digital health used but also by the physical and psychosocial work environment. It is well known that work characteristics have an important impact on employee attitudes toward the digital workplace transformation [4]. Moreover, the various effects of digitalization are likely to be interconnected. Although physicians’ perceived benefits of digitalization appear to outweigh the risks [5], there is limited knowledge about the factors affecting this balance. To achieve the benefits of digitalization, a more comprehensive understanding of this complex phenomenon related to the digitalization of physicians’ work is required.

To date, conceptual and statistical tools to analyze and illustrate such complexities have been lacking. However, the recently introduced psychological network approach offers a promising methodology to address the interplay between multiple factors in multiple areas [47]. To understand complex phenomena, it is often insufficient to only focus on how the individual components of a system function. Instead, one must also study the organization of the system’s components, which can be represented in a network. In this field of research, psychosocial, organizational, and behavioral entities are conceptualized as an interplay of social, psychological, and other components that interact in a network consisting of nodes representing observed variables and connected by edges representing statistical relationships [47-51].

### Goal of This Study

The aim of this study was to examine physicians’ perceptions of the effects of health care digitalization on their work and to analyze how these perceptions are associated with multiple factors related to work and digital health usage. With this information, it is possible to further develop digital health to meet the needs of clinical practice. In addition, the information can be used to improve the understanding of the changing nature of clinicians’ work and enable organizations to develop physicians’ orientation, promote staff empowerment and well-being, and improve the quality of care services. The research questions (RQs) were as follows:

- RQ1: How has the digitalization of health care affected the work of physicians from their perspective?
- RQ2: How are (1) the effects of digitalization (patients’ active role, preventive work, interprofessional cooperation, decision support, access to patient information, and faster consultations), (2) factors related to work (purpose of using the HIS, employment sector, and specialization status), and (3) factors related to digital health usage (EHR experience, EHR grade, participation in HIS development, and telemedicine) connected in the network structure?

## Methods

### Study Sample

The nationwide survey *EHR systems as a tool for physicians 2021* was conducted in Finland as part of the national STePS 3.0 project [52,53]. The survey method and questionnaire have been described in detail elsewhere [54]. The data were collected between January and March 2021. An invitation to participate in the web-based survey was sent by email to all physicians of working age (<65 years) who had provided their email addresses to the Finnish Medical Association (n=19,142). The register represents 90.51% (19,142/21,148 of all working age physicians who live in Finland [55]. We received responses from 4683 physicians (response rate 4683/19,142, 24.46%). A total of 53 responses were removed from the data because the respondents reported that they did not use health care information systems at all (n=43) or they did not respond to this question (n=10). The final sample included 4630 physicians (2960/4617, 64.11% women) who worked directly with patients, in administration, or both. Information on the status of clinical work was no longer included in the registry, so the number of clinically active physicians is an estimate based on several data sources and the expertise of researchers at the Finnish Medical Association. According to their analyses, the respondents were representative of the population. However, older physicians responded slightly more often than younger physicians, as did specialists. The hospital sector was also slightly overrepresented [56].

### Ethics Approval

According to Finnish legislation, a statement from the ethics committee is not required to conduct surveys on respondents’ opinions [57]. Participation in the survey on the EHR systems as a tool for physicians in 2021 was voluntary. All participants provided informed consent by choosing to participate actively in the study by answering the questionnaire.

### Context

Finland is one of the leading countries in digitalization, ranking first in a comparison of digitalization levels across European Union member states [58]. The public sector has the primary responsibility for health services, which are complemented by private sector services [59]. Private service providers deliver a quarter of all social and health services [60]. Almost half (49%) of Finnish physicians work in hospitals, a quarter (25%) in health centers, and 16% in the public sector [55].

EHRs are widely deployed across Finland [61]. As early as 2007, EHR coverage in the public sector in Finland reached 100%, and in 2017, all hospital districts achieved 100% usage intensity in conservative, operative, and psychiatric care. In 2020, 91% of private actors reported usage intensities above 90% [61]. All EHRs are integrated with national health information exchange services (Kanta services), which comprise My Kanta Pages, Prescription service, Pharmaceutical database, Patient Data Repository, and archiving of old patient data [62]. Participation in Kanta services is mandatory for all public health care providers. Private providers are also required to join Kanta services if the organization archives its clients and medical records in an electronic form [62]. In addition, there are several ancillary systems, for example, radiology and laboratory information systems, and HISs for operating rooms, intensive care units, labor and delivery, and emergency departments [63,64]. However, due to suboptimal integration solutions within the organizations, insufficient data structures in Kanta services, and barriers set by legislation, patient information is not always readily usable across different sectors, organizations, or facilities [61]. Comprehensive digital services are also made available to patients by solutions such as patient portals, access to their own data in the Kanta eArchive via My Kanta Pages, digital symptom checkers, and digital self-management guides [65]. Telemedicine with patients via video visits or chat messages, as well as self-recorded health data and monitoring services, already increased before the COVID-19 pandemic [61].

### Measurements

#### The Effects of Digitalization on Work

**Figure 1:**
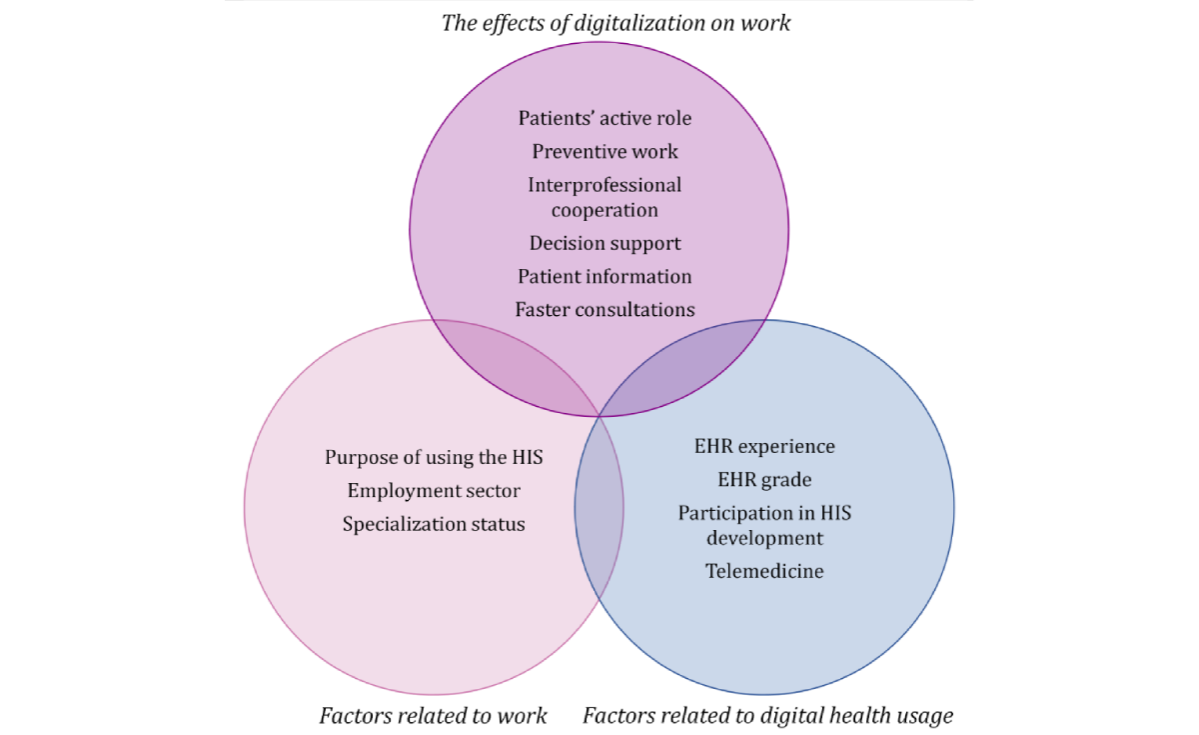
Variables used in this study. EHR: electronic health record; HIS: health information system.

All variables used are presented in Figure 1 and Multimedia Appendix 1. The measurement of the perceived effects of digitalization on work was based on the strategic focus areas of the Finnish eHealth and eSocial Strategy 2020 [66]. The survey questionnaire included 6 statements related to physicians’ experiences with digitalization. Respondents were asked how the digitalization of health care has affected their work and asked to assess the change during the last 3 years.

The 6 statements were as follows:

1. Patients have assumed a more active role in their treatment (patients’ active role).
2. Possibilities for preventive work have improved (*preventive work*).
3. Interprofessional cooperation has progressed (*interprofessional cooperation*).
4. Intelligent decision support systems support a physician’s work (*decision support*).
5. It has become easier to obtain information on patients (*patient information*).
6. Consultations with patients have become faster (*faster consultations*).

Response options were rated on a 5-point Likert scale ranging from 1 (fully agree) to 5 (fully disagree). For the analysis, the responses were reversed from 1 (fully disagree) to 5 (fully agree). Variables were recoded for the descriptive results, as (1) somewhat or fully disagree (response options 1-2), (2) neither agree nor disagree (response option 3), and (3) somewhat or fully agree (response options 4-5). In the network analysis, each statement was used separately as a continuous variable.

#### Work-Related Factors

Work-related factors included purpose of using the HIS, employment sector, and specialization status.

*Purpose of using the HIS* was assessed by asking whether respondents used the HIS (1) for patient work, (2) for administrative work, (3) for both, or (4) not at all. The variable was coded as a dichotomous variable: 0=uses not only for work with patients (response options 2-3) and 1=uses only for work with patients (response option 1). Response option 4 was coded as missing.

*Employment sector* was assessed with the following response options: (1) municipality, (2) state, (3) private (including The Social Insurance Institution of Finland [Kela]), (4) university, and (5) I am not employed. The variable was coded as a dichotomous variable: 0=public (response options 1-2) and 1=private (response options 3-4).

*Specialization status* was assessed using response options (1) not specialized, (2) in specialist training, and (3) specialized and coded as a dichotomous variable: 0=not specialized (response options 1 and 2) and 1=specialized.

#### Factors Related to Digital Health Usage

The factors related to digital health usage included EHR experience, EHR grade, participation in HIS development, and telemedicine.

*EHR experience* was assessed by asking respondents how long they had used the EHR system that they mainly use in their employment. The response options were (1) less than 6 months, (2) 6 months—less than a year, (3) 1 to 3 years, (4) 4 to 6 years, and (5) more than 6 years. The variable was coded as a dichotomous variable: 0=less than 6 months and 1=6 months or more.

*The EHR grade* was assessed by asking, “On a scale of 4 to 10 (with 4 being the lowest score and 10 being the highest score), how would you rate the EHR you mainly use?” Response options ranged from 4 to 10, including a response option of “I am not able to give a grade, or I do not wish to answer.” The variable was coded as a dichotomous variable: 0=4 to 7 (low grade) and 1=8 to 10 (high grade). The response option “I am not able to give a grade, or I do not wish to answer” was coded as missing.

*Participation in HIS development* was assessed by asking whether the respondent had participated in HIS development activities. The response options were (1) yes, some of my working time has been allocated for such development work; (2) yes, in addition to my work; and (3) no. The variable was recoded as a dichotomous variable: 0=no and 1=yes (response options 1-2).

*Telemedicine* was assessed by asking whether the respondent’s main employment involved telemedicine with patients (remote treatment by phone, chat, video contact, and other electronic contact). The response options were (1) very much, (2) much, (3) some, (4) a little, and (5) not at all. The variable was recoded as a dichotomous variable: 0=little or not at all (response options 4-5) and 1=somewhat to very much (response options 1-3).

### Statistical Analysis

Descriptive analyses were performed using SPSS (IBM SPSS Statistics 27) to characterize the sample characteristics and the variables used. Owing to nonresponse in some items, the number of observations varied in the descriptive analyses. Subsequently, network analysis was performed using mixed graphical models [67] in R Statistical Software (version 4.1.1; R Foundation for Statistical Computing 2020) to estimate the associations between the perceived effects of digitalization, work-related factors, and factors related to digital health usage. The scale-based effects of digitalization were modeled as continuous variables: patients’ active role, preventive work, interprofessional cooperation, decision support, patient information, and faster consultations. The skewed distributions of these variables were normalized using the nonparanormal transformation (huge.npn function) [68]. The following binary work-related factors were modeled as 2-level categorical variables: purpose of using the HIS, employment sector, and specialization status. The following binary digital health usage–related factors were also modeled as 2-level categorical variables: EHR experience, EHR grade, participation in HIS development, and telemedicine.

As the data contained both continuous and binary variables, we estimated the main network with mixed graphical models using the mgm package (version 1.2.12) [67]. The package estimates a network model by running regularized generalized regressions on each variable and estimating the edges associated with that variable. We provided the data, removed the missing values, and specified the type and number of levels for each variable. The regularization parameter *λ* was selected by 10-fold cross-validation, and the parameter k was set to 2 only to estimate the pairwise relationships. The computed relationships were represented in undirected graphical models [69] and visualized using the qgraph package [70]. Each variable was represented as a node in the network and pairwise connections between variables were represented as edges. The adjusted and standardized regression coefficients are denoted by b in the text. We added the strength of the dependencies by the width of the edges and information about the sign of the edges: green and blue edges indicate positive relationships and red edges indicate negative relationships. Two different colors indicating positive edges (green and blue) were used for illustration purposes.

Centrality statistics for the networks were examined to assess the relative influence of each factor in the network in terms of standardized node strength (the sum of edge weights associated with a given node) [47,71]. The predict function in mgm was used to obtain estimates of predictability for each factor. Predictability refers to the extent to which the variable can be explained by other variables included in the network [72]. Estimates are reported on a scale from 0 to 1, with 1 reflecting complete predictability. To evaluate the stability and accuracy test of the main network, the bootnet package [71] with the mgm specification was used to compute nonparametric bootstrap intervals around the estimated network edges and significance tests for edge differences using 1000 bootstrap samples.

## Results

### Characteristics of the Study Population

The characteristics of the sample are shown in Table 1. The majority (3668/4630, 79.22%) used HISs exclusively to work with patients. Most participants (3676/4614, 79.67%) worked in the public sector, and more than two-third (3134/4630, 67.69%) were specialists. For nearly half of the respondents (2177/4625, 47.07%), their work involved at least some telemedicine with patients. In total, 15.77% (727/4611) of the respondents had less than 6 months of experience with their current EHR. Nearly two-third (2974/4610, 64.51%) gave the EHR they primarily used a low rating.

**Table 1:** Characteristics of the study sample of Finnish physicians (N=4630).

Characteristic		Participants, n (%)
**Age group (years) (n=4591)**		
	<35	948 (20.65)
	35-44	1211 (26.38)
	45-54	1148 (25)
	55-64	1284 (27.97)
**Gender (n=4617)**		
	Women	2960 (64.11)
	Men	1626 (35.22)
	Other or did not want to respond	31 (0.67)
**Purpose of using the HIS^a^ (n=4630)**		
	Not only for work with patients	962 (20.78)
	Only for work with patients	3668 (79.22)
**Employment sector (n=4614)**		
	Public	3676 (79.67)
	Private	938 (20.33)
**Specialization status (n=4630)**		
	Not specialized	1496 (32.31)
	Specialized	3134 (67.69)
**EHR^b^experience (months) (n=4611)**		
	<6	727 (15.77)
	≥6	3884 (84.23)
**EHR grade (4-10) (n=4610)**		
	Low grade	2974 (64.51)
	High grade	1636 (35.49)
**Participation in HIS development (n=4601)**		
	No	3495 (75.96)
	Yes	1106 (24.04)
**Telemedicine with patients (n=4625)**		
	Little or not at all	2448 (52.93)
	Somewhat to very much	2177 (47.07)

^a^HIS: health information system.

^b^EHR: electronic health record.

### Perceived Effects of Digitalization on Work

**Figure 2:**
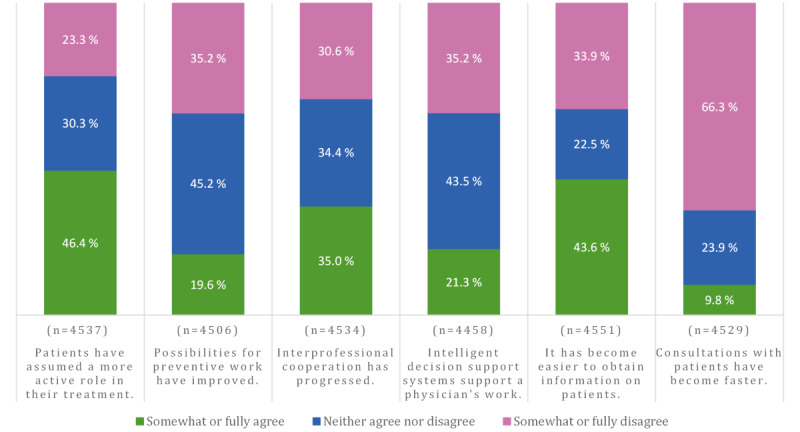
Physicians’ perceptions about the effects of digitalization on work. The scale ranged from 1 (fully disagree) to 5 (fully agree). Somewhat or fully disagree included response options 1 to 2, and somewhat or fully agree included response options 4 to 5.

The descriptive statistics of the perceived effects of digitalization are shown in Figure 2. Nearly half (2104/4537, 46.37%) of the respondents agreed that digitalization of health care has had a positive effect by giving patients a more active role in their treatment (mean 3.24, SD 0.99). This statement was rated as the most positive of all the estimated effects of digitalization on work. In addition, nearly half (1986/4551, 43.64%) of the respondents indicated that obtaining information about patients has become easier (mean 3.06, SD 1.18). The weakest positive effect of digitalization was found in consultation times (mean 2.12, SD 1.03). Only one-tenth (445/4529, 9.82%) found that consultations with patients have become faster. Almost half (2036/4506, 45.18%) of the respondents estimated that digitalization has neither increased nor decreased the possibilities for preventive work (mean 2.78, SD 0.91). Almost half (1941/4458, 43.54%) of the respondents could not state whether the CDSSs have supported their work (mean 2.77, SD 0.99). Assessments of the progress of interprofessional cooperation were evenly distributed (mean 3.01, SD 1.01).

### Network Analyses

**Figure 3:**
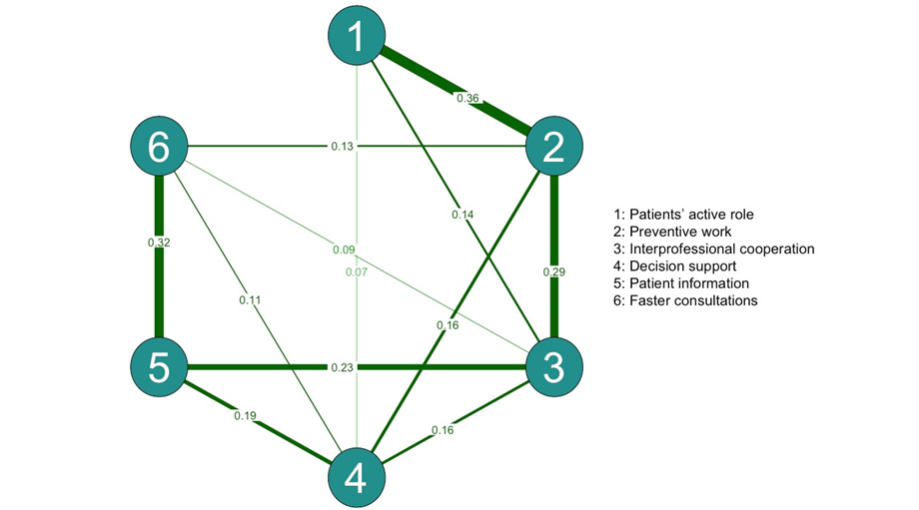
A visualized network (n=4339) of the perceived effects of digitalization on the physicians’ work. The strength of the dependency is reflected in the weight of the pairwise edge. Positive edges are shown in green.

The resulting network (Figure 3) shows the interconnections between the perceived effects of digitalization on work. In the estimated network, each node was connected to 3, 4, or 5 other nodes. The strongest associations were between the patients’ active role and preventive work (b=0.36), patient information and faster consultations (b=0.32), interprofessional cooperation and preventive work (b=0.29), interprofessional cooperation and patient information (b=0.23), and decision support and patient information (b=0.19). The other associations ranged from 0.07 to 0.16, indicating weaker interconnections.

**Figure 4:**
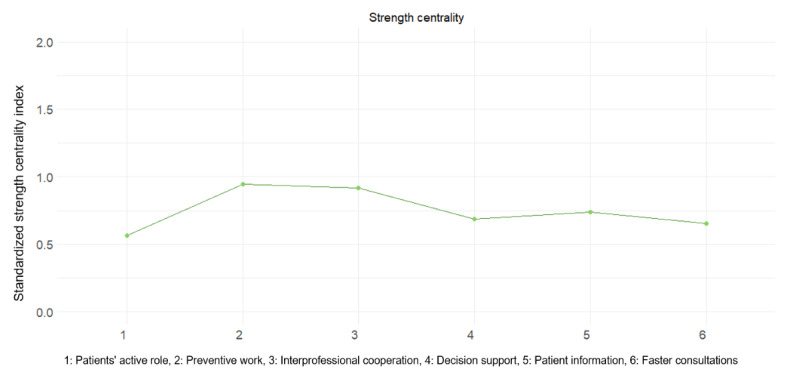
Standardized (ie, *z* scores) centrality indexes denoting node strength for perceived effects of digitalization.

According to the centrality statistics (Figure 4), the most central perceived effect of digitalization in terms of strength (ie, how strongly a variable was connected to all other nodes) was preventive work, followed by interprofessional cooperation. Patients’ active role had the lowest cumulative strength of connections to other variables.

**Figure 5:**
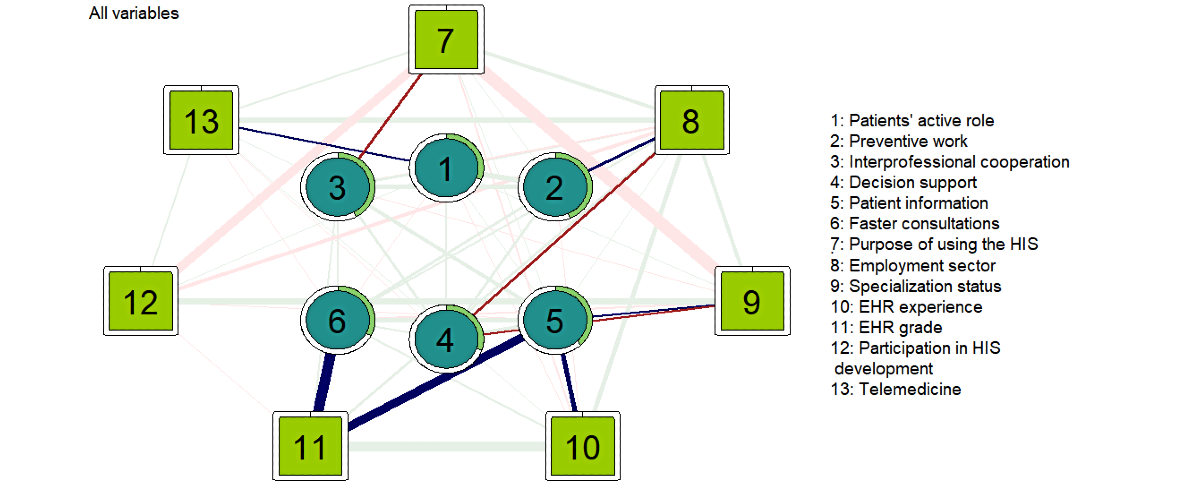
A visualized main network (n=4339) of the perceived effects of digitalization on the work and factors related to work and digital health usage. The strength of the dependency is reflected in the weight of the pairwise edge. Positive edges are shown in green and blue, and negative edges are shown in red. The green ring around each node represents its predictability. EHR: electronic health record; HIS: health information system.

The resulting main network (Figure 5) shows the connections between all 13 factors: perceived effects of digitalization on work (1-6) and factors related to work (7-9) and digital health usage (10-13). In the estimated network, the nodes were connected to 5 to 10 other nodes. The strongest direct links to effects of digitalization were with EHR grade. A higher EHR grade was associated with faster consultations (b=0.32) and facilitated access to patient information (b=0.28). At least 6 months of experience with the main EHR was associated with facilitated access to patient information (b=0.18). Using the HIS only for working with patients was negatively associated with progressed interprofessional cooperation (b=−0.16). The private sector was positively associated with improved possibilities for preventive work (b=0.16) and negatively associated with the supportiveness of CDSSs (b=−0.15). Specialization was negatively associated with the supportiveness of CDSSs (b=−0.13) and positively associated with facilitated access to patient information (b=0.13). A greater amount of telemedicine with patients was associated with a more active role of patients (b=0.12).

Pairwise connections for all visualized variables that reach a value above 0.20 are reported. There was a strong negative association between specialization status and the purpose of using the HIS (b=−0.85). A strong positive association was found between a longer experience with the main EHR and a higher EHR grade (b=0.67). There was also a strong negative association between the purpose of using the HIS and participation in HIS development (b=−0.59). In addition, being specialized was associated with participation in HIS development (b=0.38). The private sector was associated with a greater number of telemedicine services (b=0.34), longer experience with the main EHR (b=0.27), and using the HIS only for patient work (b=0.21). The predictability of each node (perceived effects of digitalization) ranged from 30.2% (decision support) to 43.6% (preventive work) of the explained variance in the continuous variables.

**Figure 6:**
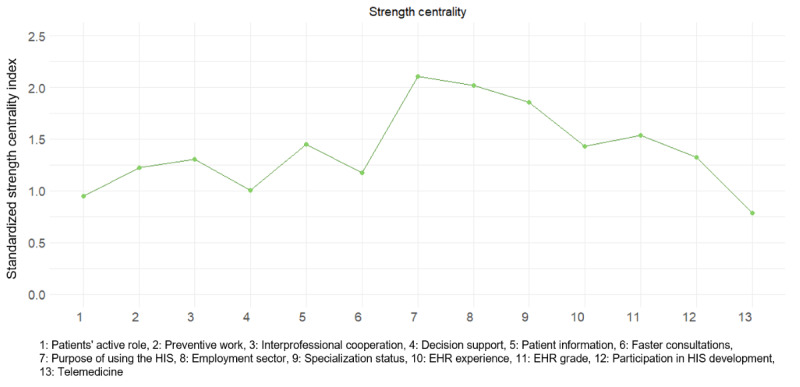
Standardized (ie, *z* scores) centrality indexes denoting node strength for each factor. EHR: electronic health record; HIS: health information system.

The work-related factors were the most central in the main network (Figure 6). In terms of strength, reflecting the overall influence in the network, purpose of using the HIS had the highest cumulative strength of connections to other variables, followed by employment sector and specialization status. Among the factors related to digital health usage, EHR grade had the highest cumulative strength, while the level of telemedicine had the lowest. Of the perceived effects of digitalization, obtaining information had the highest strength and patients’ role had the lowest.

Network stability analysis provided some large and overlapping bootstrapped CIs around the edge weights (Figure S1 in Multimedia Appendix 2). The generally large, bootstrapped CIs urge caution in interpreting the relative sizes of edges. However, correlation stability for strength centrality was 0.517, meaning that 51.7% of cases could be dropped to maintain a correlation with the original centrality greater than 0.7 with a 95% confidence. Values greater than 0.5 were considered stable. Figure S2 in Multimedia Appendix 2 shows the resulting strength-stability plot. Testing for significant differences revealed that all edges differed significantly from several other edges (Figure S3 in Multimedia Appendix 2). In addition, all node strengths differed significantly from those of at least half of the other nodes (Figure S4 in Multimedia Appendix 2).

## Discussion

### Principal Findings

On the basis of our results, nearly half of the physicians assessed that digitalization has promoted an active role for patients in their own care and easier access to patient information. However, only 1 in 10 participants felt that digitalization has had a positive impact on consultation times with patients. The network analysis highlighted the complex interdependence of several factors related to the perceived effects of digitalization on physicians’ work. The most central factors in the main network were work related: purpose of using the HIS, employment sector, and specialization status. However, the strongest direct links to the perceived effects of digitalization was with how highly physicians rated the EHR they primarily used. A higher EHR grade was associated with perceptions of faster consultations and easier access to patient information. Overall, purpose of using the HIS, employment sector, specialization status, and EHR grade seemed to be key factors that influenced how positively the effects of digitalization on work were perceived.

### The Perceived Effects of Digitalization on Work

Our results showed that nearly half of the respondents agreed that digitalization has helped patients take a more active role in their care. This finding supports the idea that digital health empowers patients to be active participants [9-11]. However, the changing role of patients may have required physicians to more actively encourage their patients to engage in digital health [73]. Patients also need a variety of skills, such as digital health literacy, to play an independent role [74]. Simultaneously, physicians are increasingly expected to assess whether patients have properly understood health information in relation to their own situation [75].

While our results show that the role of patients in their own care was seen as more active and it was associated with improved possibilities for preventive work, nearly half of the physicians estimated that digitalization had neither increased nor decreased opportunities for prevention. Although there is extensive research on the effectiveness of digital health interventions [12-17], not all digital health interventions are created equal and many lack evidence, and achieving outcomes depends on providing the right type of intervention to the right population [12]. From the point of view of the changing work of physicians, digital health prevention work may require a new kind of support for patients, which should be properly and differently targeted.

Almost half of the respondents agreed that digitalization had facilitated access to patient information. Similarly, previous studies have shown that EHRs support the bidirectional flow of patient information and facilitate information sharing among stakeholders [21]. Physicians have perceived obtaining real-time patient data as one of the benefits of EHRs [25]. Patient data sharing between hospitals has also increased [24]. According to our results, easier access to patient information was associated with faster consultation times. However, a significant majority (3003/4529, 66.31%) of the respondents disagreed that consultations with patients have become faster due to digitalization. This was clearly the lowest-rated area and indicates that the time advantage gained in obtaining the information may be lost elsewhere. Previous studies have reported, for example, increased documentation time and time spent on the computer during short-term follow-ups [18,76,77]. The initial transition to the new EHR appears to increase documentation time, but the workflow seems to improve as staff members become more familiar with the system [18]. Most physicians in this study had been using the same system for at least 6 months; thus, recent implementations were unlikely to significantly explain respondents’ time use.

Interprofessional cooperation was also connected to improved possibilities for preventive work and for more easily obtaining information on patients. The connection to improved possibilities for preventive work was presumable as interprofessional context had previously been argued to help expand a narrow interpretation of one field and promote the contribution of each member of the team [78]. Health promotion generally involves professionals with different disciplinary backgrounds, who typically also work in different sectors [79]. In this study, over one-third of the respondents estimated that interprofessional cooperation has improved. However, almost as many felt that digitalization has not influenced cooperation. Multiple studies have suggested that the implementation of EHRs [18,19] and electronic prescribing systems [20] can have negative effects on interprofessional communication. However, Chao [21] showed an increased frequency of interprofessional communication while maintaining intraprofessional communication patterns after EHR implementation. Digital health use is also shown to support interdisciplinary cooperation [22], and there are encouraging results for the use of specialists in nonspecialist telemedicine [23]. We cannot draw direct conclusions about the efficiency of collaboration, but our results suggest that digitalization is clearly changing the way professionals interact.

Nearly half of the respondents felt that digitalization had neither increased nor decreased the support physicians received from the CDSSs. However, better support from CDSSs and easier access to patient information were associated. Documenting patient information in a structured, uniform, and simple manner has been previously described as essential for electronic decision support [80]. Previous research suggests that CDSSs can support physicians [28,29,32] and improve outcomes [28,30,31], but they continue to fall short of their full potential [31]. Ford et al [81] pointed out that many previous CDSSs were not developed with the end user, practice context, or clinical workflow in mind. In addition, previous studies show that user attitudes [82] and acceptance are central to the success of CDSSs [83,84].

### The Main Network

In the estimated main network, the strongest direct links to the perceived effects of digitalization were with EHR ratings. Higher EHR grades were associated with the perceptions of faster consultations and easier access to patient information. This finding largely supports the work of other studies in this area that link EHR to accessible patient information [21,24,25]. Our network analysis also showed that longer experience with the EHR that was primarily used was associated with perceptions of facilitated access to patient information.

The network also revealed other factors directly associated with the perceived effects of digitalization on work, but these were all relatively weaker. Physicians who used the HIS only to work with patients perceived less progression of interprofessional cooperation than physicians who also used the HIS for other purposes. Physicians with administrative roles may use digital technologies and also collaborate in diverse ways. Leadership in digital health services is thought to require interprofessional and intersectoral collaboration [85]. It is also suggested that leaders in health care may generally view the effects of digitalization differently and more positively than professionals (Kaihlanen, unpublished data, June 2022).

Specialists experienced less support from CDSSs but better access to patient information compared with nonspecialists. Previous research has shown that specialists have also used CDSSs less frequently than general practitioners [86]. This could be related not only to expertise but also to the clinical work itself. General practitioners are known to find electronic medical records more useful than specialists because they are faced with a wider range of symptoms to diagnose, treat, or refer [87].

Physicians who worked in the private sector perceived better opportunities for preventive work but less support from CDSSs than physicians in the public sector. These associations may indicate the different natures of medical work in the private and public sectors. For example, specialized medical care and emergency care are provided in hospitals, and most hospitals in Finland are public sector hospitals [59]. Moreover, employers are responsible for the preventive health care of their employees, most of which is provided by the private sector [60]. The perceived lower level of support from the CDSSs may also be because the CDSSs in Finland are used less in the private sector than in the public sector [61].

Physicians who used more telemedicine with patients felt that digitalization has promoted the active role of patients more than those who barely used telemedicine or did not use it at all. Previous studies have also shown that telemedicine requires patient engagement but also encourages patients to take more responsibility for their own care [88,89]. In addition, physicians who have used telemedicine are more likely known to perceive the potential benefits of telemedicine than physicians who do not use telemedicine with patients [90].

Work-related factors (purpose of using the HIS, employment sector, and specialization status) were found to be the most central factors in the network. Our results suggest that physicians who use HISs primarily for patient work participate less in development work than physicians who also have administrative responsibilities. Participation is known to provide an important sense of control over one’s work [27], and physician-initiated improvements to EHR systems have also been found to be useful [91]. The estimated network also showed that specialized physicians were more likely to participate in development work than physicians who were not specialized or were still in training. One possible explanation for this is that those with specialization are in demand for development work because their expertise is in a narrow area of medicine and they receive recognition for their esoteric skills and knowledge [92].

Working in the private sector was associated with a greater amount of telemedicine, longer experience with the main EHR, and the use of HISs only for patient work. An earlier study also found differences among practices, hospitals, and academic medical centers in the use of telemedicine with patients [93], and private hospitals have been more successful than public hospitals in adopting telemedicine [94]. Physicians in the private sector also had more experience with the primary EHR brand used than those in the public sector. Longer experience with the EHR was associated with higher EHR ratings. Shorter experience may indicate new staff members or recent EHR implementation. Several previous studies have shown that physicians are less satisfied after implementing a new EHR [43,44] primarily because of increased workflow disruptions [45]. Significantly more disruptions are noted during the transition period of approximately 6 months, after which the situation recovers [46]. Overall, the results suggest that the employer sector plays an important role in indirectly influencing how physicians view the effects of digitalization through factors related to telemedicine, experience with using EHRs, and the purpose of using HISs.

### Limitations

This study has several limitations. First, the psychometric properties of the measure of the perceived effects of digitalization on physicians’ work have not been tested previously. However, the statements are based on the Finnish eHealth and eSocial Strategy 2020 and describe the effects of digitalization on the work of physicians from the perspective of the focus areas included in the strategy. Moreover, the items were planned by a large team of experts who have been working on the digitalization of physicians’ work for a long time. The content and wording of the measures were pilot tested and evaluated by 2 physicians. On the basis of their feedback, minor revisions were made before the survey was disseminated. Contrary to many previous studies that have focused on the negative ramifications of digitalization on physicians’ work [40,95], our questions focused on aspects that can be considered to be positive in nature. Thus, the results may have been different if we would have focused on the negative ramifications of digitalization on the work of physicians.

Second, we used the Likert scale variables as continuous variables in the network, but this has been successfully practiced in other studies of mgm network modeling [eg, 96]. Third, as our data were cross-sectional, the directionality of the observed relationships was not established. Some factors may precede others but some may both contribute to and be influenced by other factors in the network. The network analyses suggest that incorporating factors measured over time, or studies that rely on longitudinal structural model tests, could provide the confirmatory evidence needed [47]. In addition, the response rate was relatively low (24.46%), indicating a higher likelihood of nonresponse bias. However, the sample size was large, and it was well representative of the target population [56]. Finally, Finland is one of the pioneers of the digitalization of health care, and tax-funded universal health care is available to all residents [97]. Therefore, caution should be exercised when generalizing our findings to countries with other types of health care systems or information communication technology infrastructure. However, digitalization is advancing at a rapid pace, and all physicians and health care organizations should be prepared for future changes.

### Conclusions

Our results suggest that, from the physicians’ perspective, digitalization has improved the active role of patients and the patient information flow, while consultation times with patients have not become faster. Further studies are needed to examine where the potential time benefit of accessible patient information is lost and how the potentially increased documentation time affects the quality of care. Physicians’ work should be organized so that the time spent on the computer is not out of the time spent with patients during appointments.

The network highlights that several factors have a complex relationship with the perceived effects of digitalization on physicians’ work. The EHR system used appears to be critical for easier access to patient information and faster consultations. Therefore, it seems that a high-quality EHR system is important for the promotion of smooth clinical practice. Thus, organizations could benefit by investing in a well-functioning EHR and the factors that influence its successful use.

In addition, the physician’s work (patient or administrative), employer sector, and career stage may play an important background role and influence many other factors that affect the success of digital interventions and their implementation. Thus, it would be advisable for organizations to involve specialists and nonspecialists equally when developing new digital tools or processes in their work. Moreover, some benefits of digitalization seem to be sector specific. Therefore, the specific context and intended use should be considered when developing and implementing digital health. Sectors can also learn from each other; for example, when and how to use telemedicine with patients, how to use digital health in health promotion, and how to benefit from CDSSs in practice. Thus, the information flow and exchange between sectors should be improved.

Overall, a more comprehensive view is needed when assessing the impact of digitalization on specific work environments and work processes. Digitalization of work and related factors should also be considered when orienting physicians. When new digital health is introduced, training on changing work processes should be provided. This would be a priority in addition to technical training. The introduction of digital health is not only a technological project but also a project that changes existing work practices and the professionals’ work environment.

## Appendix

Multimedia Appendix 1 Variables used in this study.

Multimedia Appendix 2 Stability analyses.

## Abbreviations

CDSS: clinical decision support system
EHR: electronic health record
HIS: health information system
RQ: research question

## Data Availability

The data sets generated and analyzed during this study are available from the corresponding author PS upon reasonable request after approval by the study group and the Finnish Medical Association.
