# Supplementary material for: The Interplay of Work, Digital Health Usage, and the Perceived Effects of Digitalization on Physicians’ Work: Network Analysis Approach
Source: J Med Internet Res. 2022 Aug 17;24(8):e38714. doi: 10.2196/38714 (PMC9434392; doi:10.2196/38714)
Supplement: Multimedia Appendix 1 [file jmir_v24i8e38714_app1.docx]

**Multimedia Appendix 1.**

## Variables used in this study

### The effects of digitalization on work

Electronic health care services have increased. EHR systems have been used for a long time but, in addition, patients are increasingly often offered electronic services, such as self-care services, teleconsultation, appointment scheduling, and services for recording and viewing their data.

How has such digitalization of health care affected your work? Please assess the change during the past three years.

1. Patients have assumed a more active role in their treatment.
2. Possibilities for preventive work have improved.
3. Interprofessional cooperation has progressed.
4. Intelligent decision support systems support a physician’s work.
5. It has become easier to obtain information on patients.
6. Consultations with patients have become faster.

Response options:

1. Fully agree
2. Somewhat agree
3. Neither agree nor disagree
4. Somewhat disagree
5. Fully disagree

### Work-related factors

#### Purpose of HIS use

I use health care information systems

- 1. for my work with patients
  2. for administrative work
  3. for both my work with patients and administrative work
  4. I don’t use health care information systems at all

#### Employment sector

Main employment sector

1. Municipality
2. State
3. Private (incl. The Social Insurance Institution of Finland (Kela))
4. University
5. I am not employed

#### Specialization status

Stage of specialization

- 1. Not specialized
  2. In specialist training
  3. Specialized

### Factors related to digital health usage

#### EHR experience

For how long have you used the system (EHR system you MAINLY use in your employment)?

- 1. Less than 6 months
  2. 6 months - less than a year
  3. 1 - 3 years
  4. 4 - 6 years
  5. More than 6 years

#### The EHR grade

On a scale of 4 to 10 (with 4 being the lowest score and 10 being the highest score) how would you rate the EHR you mainly use?

- 1. 10
  2. 9
  3. 8
  4. 7
  5. 6
  6. 5
  7. 4
  8. I am not able to give a grade, or I do not wish to answer

#### Participation in HIS development

Have you participated in information systems development work?

- 1. Yes, some of my working time has been allocated for such development work
  2. Yes, in addition to my work
  3. No

#### Telemedicine

Does your main employment involve teleconsultation with patients (remote treatment by phone, chat, video contact, other electronic contact)?

- 1. Very much
  2. Much
  3. Some
  4. A little
  5. Not at all
