# Supplementary material for: The Interplay of Work, Digital Health Usage, and the Perceived Effects of Digitalization on Physicians’ Work: Network Analysis Approach
Source: J Med Internet Res. 2022 Aug 17;24(8):e38714. doi: 10.2196/38714 (PMC9434392; doi:10.2196/38714)
Supplement: Multimedia Appendix 2 [file jmir_v24i8e38714_app2.docx]

**Multimedia Appendix 2.**

## Stability Analyses

Results of *bootnet* stability analyses are summarized in the text, with corresponding figures below.


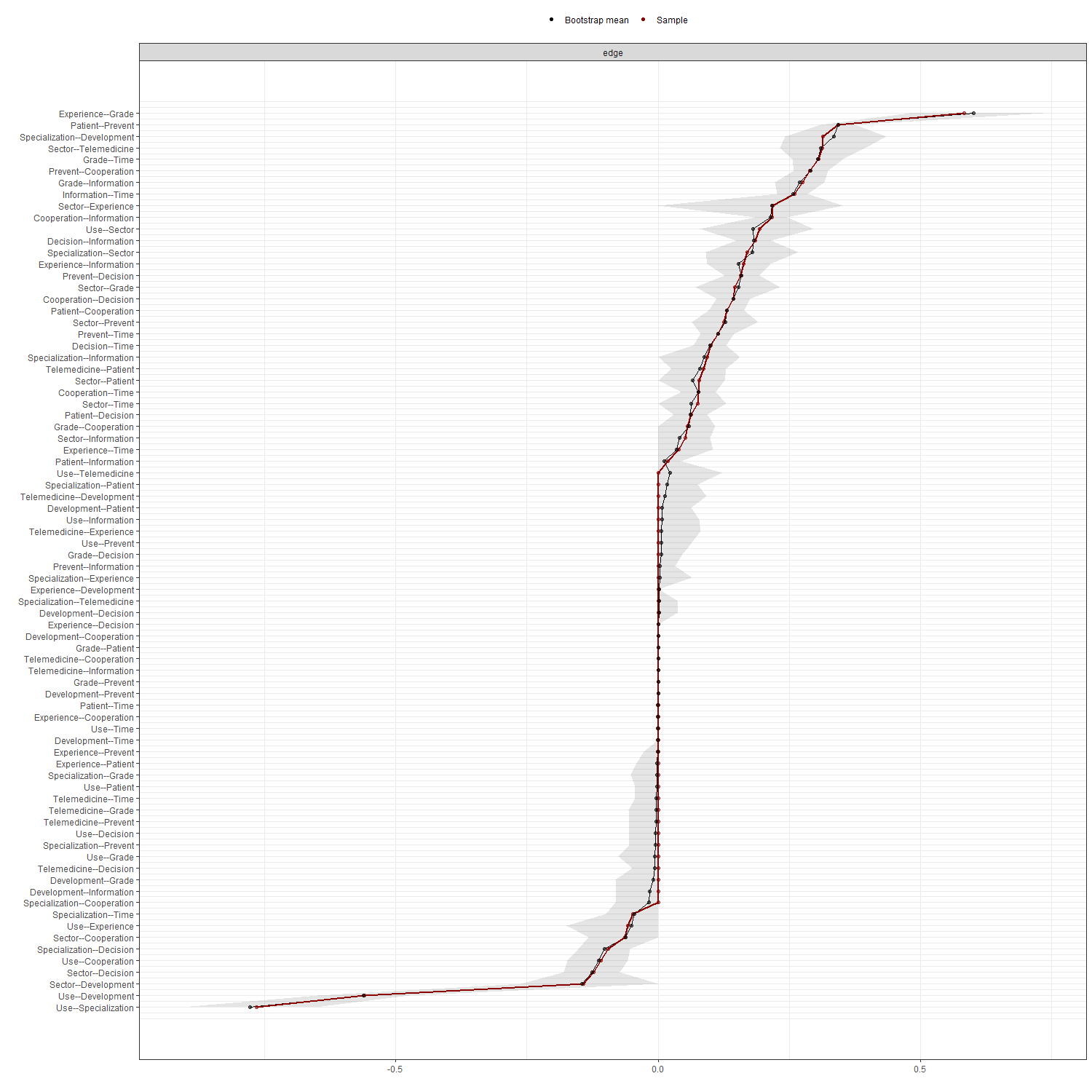


Figure S1. Bootstrapped CIs of the estimated edge weights for the main network. The red line shows the sample values, the black dots show the bootstrap averages, and the gray area shows the bootstrapped CIs. Each horizontal line represents an edge of the network, ordered from highest to lowest edge weight.


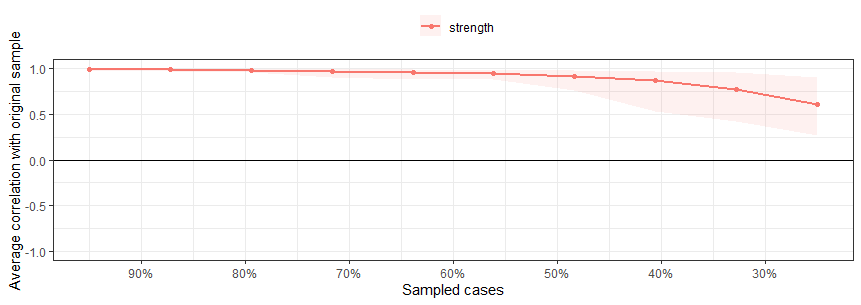


Figure S2. Average correlations between the strength of networks where persons were dropped from the sample and the original sample. The lines show the mean values and areas indicate the range between the 2.5th and the 97.5th quantile.


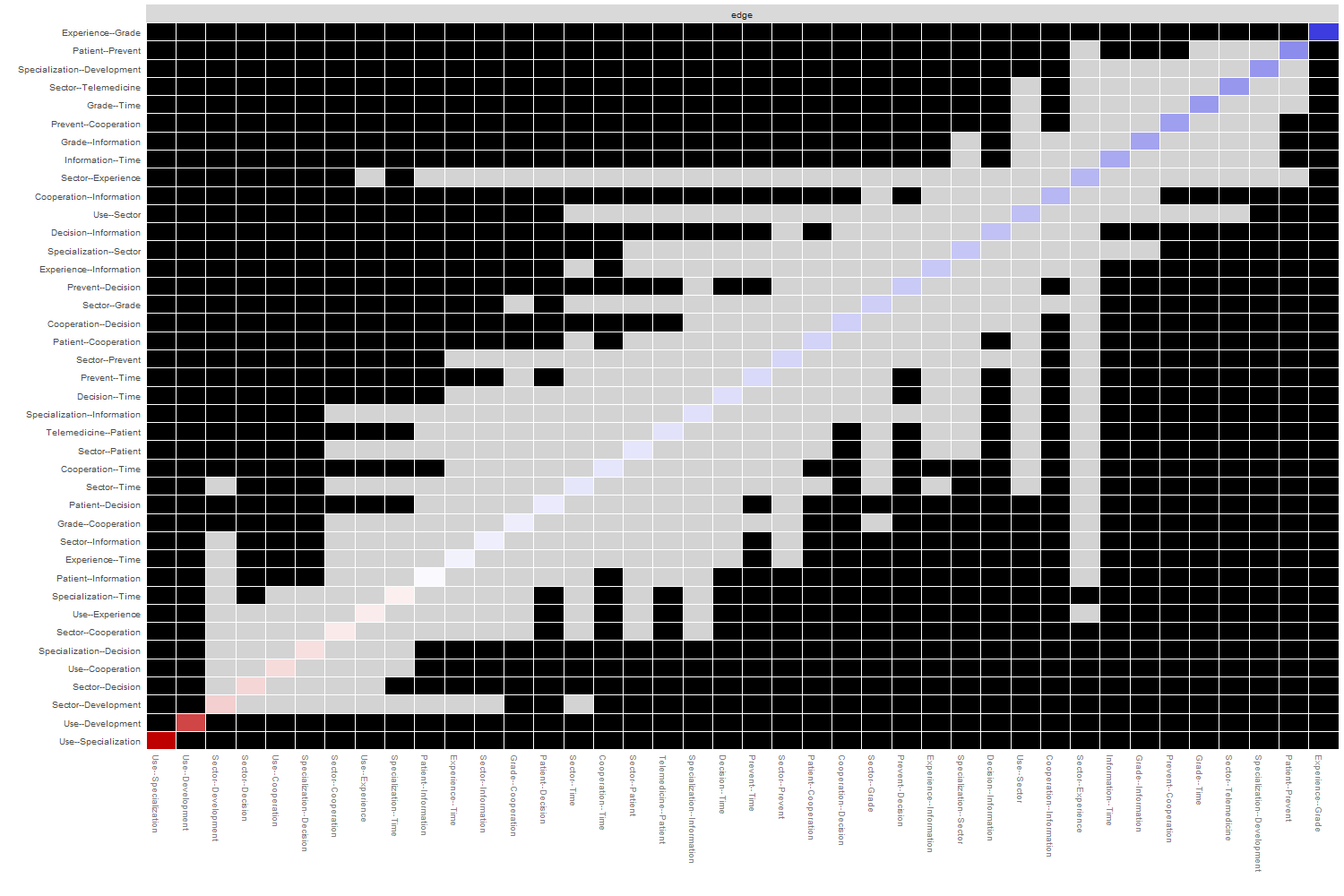
Figure S3. Bootstrapped difference tests (α = 0.05) between edge weights in the network. Gray boxes indicate edges that are not significantly different from each other, and black boxes edges that are significantly different from each other.


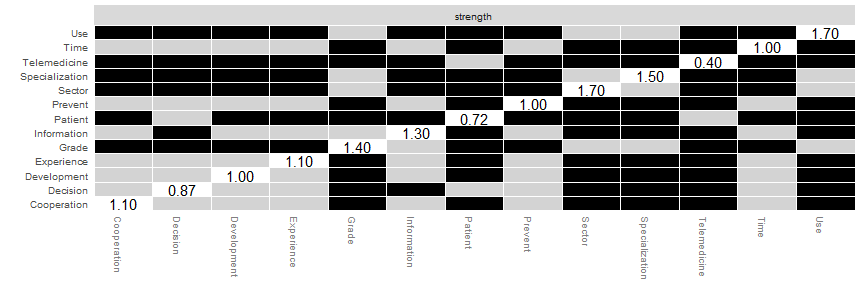


Figure S4. Bootstrapped difference tests (α = 0.05) between node strength of the 13 factors. Gray boxes indicate nodes that are not significantly different from each other, and black boxes nodes that are significantly different from each other. White boxes indicate the value of the node strength.
